# What are genome-wide association studies telling us about B-cell tumor development?

**DOI:** 10.18632/oncotarget.169

**Published:** 2010-09-11

**Authors:** Amy L Sherborne, Richard S Houlston

**Affiliations:** Section of Cancer Genetics, Institute of Cancer Research, Sutton, Surrey, SM2 5NG, UK

## Abstract

It has long been speculated that common genetic variation influences the development of B-cell malignancy, however until recently evidence for this assertion was lacking. The advent of genome-wide association studies (GWAS) has allowed the search for this class of susceptibility allele to be conducted on a genome-wide basis. Recent GWAS of chronic lymphocytic leukemia (CLL) and acute lymphoblastic leukemia (ALL) have identified novel disease genes for CLL and ALL and underscore the importance of polymorphic variation in B-cell development genes as determinants of leukemia risk.

## INTRODUCTION

The identification of cancer susceptibility genes has provided for a greater understanding of the mechanisms of tumor biology. Furthermore, genetic associations are likely to prove increasingly valuable via the functional links they reveal, either endorsing current etiological hypotheses or suggesting novel ones that merit testing via gene-environment specific hypotheses.

While the familial clustering of B-cell malignancies was well recognized over forty years ago and a strong association between HLA and Hodgkin lymphoma (HL) risk established in 1967 [1], it is only recently that concerted efforts have been directed to understanding inherited susceptibility to hematological malignancy.

Here we discuss the impact findings from genome-wide association studies (GWAS) are having on our understanding of B-cell chronic lymphocytic leukemia (CLL) and acute lymphoblastic leukemia (ALL) development.

## INHERITED GENETIC SUSCEPTIBILITY TO CLL AND ALL

B-cell CLL (MIM 151400) accounts for ~25% of all leukemia and is the most common form of adult lymphoid malignancy in Western countries. Evidence for inherited genetic predisposition to CLL is provided by a seven-fold elevated risk of CLL seen in first-degree relatives of CLL patients [2, 3]. Genome-wide linkage scans of CLL families have, however, consistently failed to identify a high risk disease locus for CLL locus making a polygenic model of inherited predisposition based on the co-inheritance of multiple low-risk alleles more likely [4, 5].

Acute lymphoblastic leukemia is the major pediatric cancer in economically developed countries; precursor B-cell (BCP-ALL; MIM 613065) accounting for ~70% of childhood ALL. In contrast to CLL, evidence for a familial risk to ALL is weak. Data from the Swedish family-cancer database does, however, lend some support to an excess risk in relatives of patients, independent of the high concordance in monozygotic twins [6], which has a non-genetic, in-utero explanation. Although rare (<5% of ALL), direct evidence for inherited genetic susceptibility is provided by the high risk of ALL associated with Bloom's syndrome, neurofibromatosis, ataxia telangiectasia and constitutional trisomy 21. While evidence linking an environmental exposure to risk of childhood ALL has largely been inconsistent, epidemiological data for an infectious etiology is persuasive, albeit indirect [7]. Implicit in a model of ALL having an infectious etiological basis is that ALL represents a rare sequelae [7] of infection with germline variation influencing host response.

## CANDIDATE GENE POLYMORPHISM STUDIES

It has long been speculated that common polymorphic variation contributes to the susceptibility of hematological malignancy but evidence for common low risk alleles has emerged only recently. Numerous associations have been proposed from candidate gene analyses conducted throughout the past 30 years. Despite much research, no definitive susceptibility alleles have been unequivocally identified through these studies. As with many other diseases, positive associations have been reported for various polymorphisms but few of the initial positive results have been replicated in subsequent studies. The inherent statistical uncertainty of case-control studies involving just a few hundred cases and controls seriously limits the power of such studies to reliably identify genetic determinants conferring modest but potentially important risks. Furthermore, without a clear understanding of the biology of predisposition, the definition of suitable genes for the disease is inherently problematic making an unbiased approach to loci selection highly desirable.

## THE ADVENT OF GENOME-WIDE ASSOCIATION STUDIES

Recently, GWAS have proved a powerful approach for the identification of common, low-penetrance loci for cancer without prior knowledge of location and function. Most of these GWA studies are based on tag SNPs which capture a high proportion of common variation in the genome through linkage disequilibrium (LD). This approach is unbiased and does not depend upon prior knowledge of function or presumptive involvement of any gene in disease causation. Moreover, this strategy avoids the possibility of missing the identification of important variants in hitherto unstudied genes.

## GENOME-WIDE ASSOCIATION STUDIES OF CLL AND ALL

To identify common disease causing alleles for CLL and ALL we have recently conducted GWA studies of these diseases using a two-stage strategy [8-11]. In the first stage we compared the frequency of ~300,000 tag SNP genotypes in a discovery set of cases and controls and the SNPs showing the strongest associations were subsequently genotyped in multiple independent case-control series. The two-stage strategy is highly efficient and use of multiple independent case-control series guarded against false positives being reported. Only P-values of ~1.0x10^−7^ are conventionally regarded as providing evidence of significance at the genome-wide level.

To date we have identified 10 novel CLL risk loci at 2q13 (rs17483466), 2q37.1 (rs13397985, SP140), 6p25.3 (rs872071, IRF4), 11q24.1 (rs735665), 15q23 (rs7176508), and 19q13.32 (rs11083846, PRKD2) 2q37.3 (rs757978), 8q24.21 (rs2456449), 15q21.3 (rs7169431) and 16q24.1 (rs305061, IRF8) [8, 10] and four risk loci for ALL at 7p12.2 (IKZF1, rs4132601), 10q21.2 (ARID5B, rs7089424) and 14q11.2 (CEBPE, rs2239633) [9, 11, 12]. Intriguingly, excluding *CDKN2A*, none of the genes implicated by these GWA scans have previously been evaluated in targeted association studies, emphasizing that the candidate gene approach was severely limited by inadequate knowledge of tumor biology.

## IMPACT OF LOW-RISK SUSCEPTIBILITY ALLELES TO INDIVIDUAL RISK OF CLL AND ALL

While the risks of CLL and ALL associated with each of the 14 SNPs we identified are modest, relative risks 1.2-1.7 per allele (Table 1) their respective contribution to disease incidence is significant as a high proportion of the population are carriers of these risk alleles. Furthermore, the risk increases with increasing numbers of variant alleles and for the 2% of the population who possess 13 or more risk alleles the risk of CLL is increased ~8-fold [8]. The present data, however, provide only crude estimates of the overall effect on susceptibility attributable to variation at these loci. The effect of the actual common causal variant responsible for the association, once identified, will typically be larger. Furthermore, many of the loci may carry additional risk variants, potentially including low-frequency variants with larger influences on disease risk.

**Table 1:** CLL and ALL susceptibility loci identified through genome-wide association studies [8-12]

	Chr	Position	SNP	Gene	Risk allele	RAF	OR
CLL	2q13	111,513,929	rs17483466		G	0.20	1.39
	2q37.1	230,799,467	rs13397985	*SP140*	G	0.19	1.41
	2q37.3	242,019,774	rs757978	*FARP2*	A	0.15	1.39
	6p25.3	356,064	rs872071	*IRF4*	A	0.54	1.54
	8q24.21	128,262,163	rs2456449		G	0.36	1.26
	11q24.1	122,866,607	rs735665		A	0.21	1.45
	15q21.3	54,128,188	rs7169431		A	0.12	1.36
	15q23	67,806,044	rs7176508		A	0.37	1.37
	16q24.1	84,533,160	rs305061		C	0.33	1.22
	19q13.32	51,899,494	rs11083846	*PRKD2*	A	0.22	1.35
ALL	7p12.2	50,438,098	rs4132601	*IKZF1*	C	0.28	1.69
	9p21.3	21,974,661	rs3731217	*CDKN2A CDKN2B*	G	0.15	1.41
	10q21.2	63,422,165	rs7089424	*ARIDB5*	C	0.34	1.65
	14q11.2	22,658,897	rs2239633	*CEBPE*	G	0.52	1.34

Abbreviations: RAF, risk allele frequency; OR, odds ratio

Notes: SNP positions based on build 36.3 Risk allele frequency in Europeans unless otherwise stated Odds ratios quoted are per allele OR from primary or largest study

## IMPLICATIONS OF GWAS FINDINGS TO OUR UNDERSTANDING OF B-CELL TUMOR DEVELOPMENT

The SNP genotyped in GWAS are not generally candidates for causality, and enumeration of the causal variant at a specific locus can poise a significant challenge. While fine-mapping and resequencing is required to identify functional variant(s) the associations identified for CLL and ALL implicate a number of genes in the etiology of B-cell tumors.

**Figure 1:**
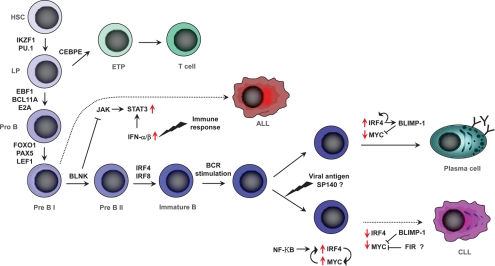
Genes regulating lymphoid development

**Figure 2:**
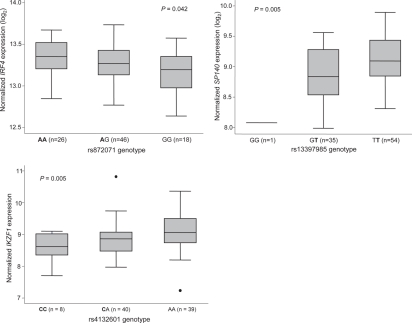
Relationship between lymphocyte mRNA expression levels of IRF4 and rs872071 genotype, SP140 and rs13397985 genotype, and IKZF1 and rs4132601 genotype Risk alleles emboldened [10, 11]

On the assumption of cis-effects GWAS findings implicate variation in *SP140*, *IRF4, PRKD2* and *IRF8* in CLL development. *IRF4* is a strong candidate for a CLL susceptibility gene *a priori* being a key regulator of lymphocyte development, and proliferation (Figure 1) [13-15]. Through interaction with transcription factors including PU.1, IRF4 controls the termination of pre-B-cell receptor signaling and promotes the differentiation of pro-B cells to small B-cells. Furthermore, via BLIMP1 and BCL6, IRF4 controls the transition of memory B-cells, thought to be the precursor cell type for CLL, to plasma cells. A model of disease etiology based on the causal variant influencing risk by arresting transition of memory B-cells through decreased *IRF4* expression is supported by a relationship between risk genotype and mRNA expression level; expression being significantly associated with genotype in a dose-dependent fashion with lower levels being associated with risk alleles [10] (Figure 2). IRF8 is also part of the transcriptional network which governs B-cell lineage, specification and differentiation, regulating α- and β-interferon response, immunoglobulin rearrangement and regulation of germinal center reaction [16]. Over-expression of *IRF8* in differentiated B-cell progenitors is associated with growth inhibition and apoptosis with *IFR8* knockout mice displaying reduced numbers of pre- and pro-B cells and increased numbers of myeloid cells.

*SP140/LYSp100B* is the lymphoid-restricted homolog of *SP100* expressed in all mature B cells and plasma cell lines, as well as some T-cells. *SP100* is a major mediator of EBV-encoded nuclear antigen leader protein co-activation which is important for establishment of latent viral infections and B-cell immortalization [17]. Since *SP140* expression is implicated in host response to immunodeficiency virus type 1 [18] it is possible that *SP140* genotype influences CLL risk by affecting response to antigenic challenge.

While a role for *PRKD2* (Protein Kinase D2) in CLL is limited, low levels of *PRKD2* expression and autophosphorylation have been reported to be a feature of a number of B-cell tumors including mantle cell and Burkitt's lymphoma, and ~50% of CLL/small lymphocytic lymphomas [19].

A number of the SNPs associated with CLL risk map to non-coding regions of the genome, most notably those which define the 8q24.21 association. GWAS of other cancers have shown that the 128–130 Mb genomic interval at 8q24.21 harbors multiple independent loci with different tumor specificities which are distinct from the CLL association [20-25]. The 8q24.21 region to which these cancer associations map is bereft of genes and predicted transcripts. The colorectal and prostate cancer SNP rs6983267 has been shown to affect TCF4 binding to an enhancer for *MYC*, providing a mechanistic basis for an 8q24.21 association [26, 27]. If the 8q24.21 CLL locus influences risk through a similar cis-effect on differential *MYC* expression, the association is intriguing because *MYC* is a direct target of *IRF4* in activated B-cells.

The strongest association signal for ALL in our GWAS and a contemporaneous GWAS conducted by other researchers was attained at 7p12.2 with rs4132601, which maps to the 3' region of the Ikaros family zinc finger 1 (*IKZF1*) gene. Ikaros proteins are master regulators of lymphocyte development (Figure 1) and differentiation plays a pivotal role in CD4 versus CD8 T-cell lineage commitment decisions [28, 29]. In homozygous mutant mice deleted for the N-terminal zinc finger DNA binding domain of *IKZF1* loss of expression leads to arrest of lymphocyte development at its earliest recognizable stage followed by rapid development of leukemia. The observation of a strong relationship between rs4132601 genotype and *IKZF1* mRNA expression level in EBV-transformed lymphocytes is thus consistent with a model in which the causal variant influences risk by impacting on early B-cell differentiation [11] (Figure 2).

The association at 10q21.2 implicates the AT rich interactive domain 5B (*ARID5B*) gene in the etiology of ALL. While ARID5B has not been extensively studied evidence for *ARID5B* having a role in defining B-cell lineage is supported by data from homozygous knockout mice, which display decreased bone marrow cellularity and reduced numbers of B-cell progenitors [30].

The 14q11.2 association with ALL annotates the gene encoding CCAAT/enhancer-binding protein, epsilon (*CEBPE*). *CEBP* is a suppressor of myeloid leukemogenesis. *CEBPE* and along with other *CEBP* family members is occasionally targeted by recurrent IGH translocations in BCP-ALL [31] suggesting opposing functions of CEBP dysregulation in myeloid and lymphoid leukemogenesis and a role in susceptibility to ALL.

The primary impact of variation defined by the 7p12.2, 10q21.2 and 14q11.2 risk variants on ALL risk is for B-cell disease. The 10q21.2 (*ARID5B*) risk association however, appears to be highly selective for the subset of BCP-ALL with hyperdiploidy [11, 12].

The region of LD defining the 9p21.3 association encompasses the *CDKN2A* and *CDKN2B* tumor-suppressor genes and the noncoding antisense RNA encoded by *CDKN2BAS*. *CDKN2A* encodes both p16 (INK4A), a negative regulator of cyclin-dependant kinases, and p14 (ARF1), an activator of p53. *CDKN2A* and *CDKN2B* are frequently inactivated in multiple hematological malignancies. Moreover, mono- or biallelic deletion of *CDKN2A* is one of the most frequent genetic events in both childhood BCP and T-ALL. Perhaps not surprisingly the association between 9p21.3 risk genotype and ALL is generic and not confined to a specific form of ALL.

## CONCLUSIONS AND FUTURE CHALLENGES

GWAS have provided the first unambiguous evidence that common genetic variation contributes to the risk of developing CLL and ALL and implicate genes involved in transcriptional regulation and differentiation of B-cell progenitors as the biological basis of predisposition to B-cell malignancy. Furthermore, their identification provides novel insight into disease causation of these two major haematological malignancies. The cell lineage of CLL coupled with the reciprocal familial risks between CLL and other B-cell LPDs including HD and non-Hodgkin lymphoma suggests that the variants may also influence the risk of other related B-cell LPDs. As the risk variants identified for CLL account for <10% of the familial risk of CLL, the prospects for identifying additional variants for this disease through additional GWAS should be high, and such studies are likely to provide further insights in the biology of B-cell tumors.
